# COVID-19 vaccine hesitancy and willingness to pay: Emergent factors from a cross-sectional study in Nigeria

**DOI:** 10.1016/j.jvacx.2021.100112

**Published:** 2021-09-03

**Authors:** Obi Peter Adigwe

**Affiliations:** National Institute for Pharmaceutical Research and Development, Plot 942, Cadastral Zone C16, Idu Industrial District, P.M.B. 21 Garki, Abuja, FCT, Nigeria

**Keywords:** Immunisation, COVID-19, Hesitancy, Vaccine

## Abstract

**Introduction:**

Prior to the COVID-19 pandemic, it took at least several years to develop vaccines for prevention of infectious diseases. The COVID-19 vaccine is the first to be developed within a period of one year. The expediency associated with the development of the COVID-19 vaccine has however been hampered by vaccine hesitancy and other relevant factors that could influence consequent immunisation. This study aimed at investigating factors associated with vaccine hesitancy and willingness to pay for COVID-19 vaccination.

**Methods:**

A cross-sectional approach was used to undertake online and physical data collection with a validated questionnaire.

**Results:**

A total of 1767 valid responses were received, female participants were in the minority (42.2%), majority (54.9%) of the study participants were between the ages of 18 and 30 years, and more than half (53.8%) of the participants were educated up to first degree level. Slightly above half (52.9%) of the study participants indicated that they were worried about side effects that may be associated with COVID-19 vaccines, and this may likely prevent them from taking the vaccine. A strong majority (85.1%) of the study participants indicated that COVID-19 vaccine should be administered at no cost to citizens. Only a quarter (26%) of the participants were willing to pay a fee for COVID-19 vaccination. Also, older participants and those that had been previously infected with COVID-19 were more likely to pay for COVID-19 vaccination.

**Conclusion:**

This study provides critical insights which could influence immunisation efforts during the pandemic. An early understanding of population perceptions of the COVID-19 vaccine can be invaluable in designing successful campaigns. This is even more critical, given supply limitations, access issues and vaccines’ inequity occasioned by the international scramble.

## Introduction

In the past one year, the COVID-19 pandemic has emerged as a major concern for global public health and socioeconomic development. This is mainly due to its considerable health sector impact combined with the deleterious effects it has been associated with in societies and economies worldwide [1], [2]. As vaccines have been identified as a key intervention, it is necessary for governments to expedite actions in ensuring large-scale, equitable access and distribution of COVID-19 vaccine, so as to promote sustainable public health solutions [3]. Several factors however exist which threaten the utility of this public health tool. Vaccine hesitancy has emerged as a global challenge and there is increasing worldwide concern about a general non-acceptance of vaccines [4]. In developing health system capacities and strategies necessary to combat the pandemic, it is important to undertake a robust and comprehensive engagement with factors likely to enhance the uptake of COVID-19 vaccines.

Currently these efforts are at risk, with anti-vaccination activists campaigning in multiple countries against the need for vaccines. Some of them even deny the existence of COVID-19 [5]. The misinformation being spread across various platforms has the potential to negatively influence the acceptance of the newly developed COVID-19 vaccines [6]. The accelerated development of several COVID-19 vaccines has also heightened public anxieties and could further compromise acceptance of the new interventions [7]. The pervasive misinformation alongside the associated vaccine hesitancy could limit the response to the current crisis as well as exacerbate relevant global public health risks. For instance, widespread misinformation in communities can prevent the attainment of relevant immunisation uptake thresholds associated with herd immunity, thereby increasing the risk of outbreak of vaccine-preventable diseases [8]. Another factor that has emerged as critical to vaccines’ acceptability as well as to immunisation implementation policies, is the willingness of the population to pay for the intervention. Evidence from the extant literature identified that willingness to pay for vaccines was a critical indicator of public perception and demand [9], [10]. Thus, the introduction of a new vaccine may require investigating public willingness to pay for it. Willingness to pay for vaccination varies depending on vaccine type and severity of disease [11]. The recognition of this important factor has therefore emerged as an invaluable decision making tool for policymaking in vaccination and immunisation [12]. The criticality of engaging with this tool is even more important in resource scarce settings such as Nigeria, especially given the international scramble orchestrated by high income nations that has resulted in inequitable distribution in vaccines access [13].

In reducing hesitancy and improving vaccine uptake, there is need for context-specific research explicitly aimed at identifying factors associated with the phenomenon [14]. A literature search revealed that no COVID-19 related study has robustly explored factors associated with vaccine hesitancy whilst also assessing a willingness to pay for emergent vaccines. It is against this backdrop that this study aimed at investigating factors associated with vaccine hesitancy and willingness to pay for COVID-19 vaccination in Nigeria.

## Methods

A cross-sectional survey was undertaken in Abuja, Nigeria in the month of January 2021. The data collection tool employed was an English language questionnaire that had undergone face and content validation by an expert panel. The questionnaire was pretested by administration to 21 participants, the feedback received did not warrant any major change. Data were collected using online and physical methods of questionnaire administration. Snowball sampling strategy was used during the online data collection process [15], [16]. Online participants were recruited using Whatsapp platform, questionnaire link was sent to various groups comprising of Abuja residents. Respondents were also asked to forward the link to their friends residing in Abuja. Participants who clicked on the link were directed to Google forms where appropriate instructions about filling the questionnaire were given. Hard copies of questionnaire were administered physically using random sampling strategy [17], in order to ensure the inclusion of individuals that lack access to the internet. Strategic locations which include motor parks, worship centres, and corporate offices were visited to recruit participants for the paper-based data collection.

Ethical approval was obtained from Institutional Review Board of National Institute for Pharmaceutical Research and Development before the commencement of data collection. Participation in the study was voluntary as informed consent was sought prior to the administration of the questionnaire. Following the importation of data collected into Statistical Package for Social Sciences software version 25, descriptive statistics were carried out. Association between variables were tested using chi square. A *p-*value of 0.05 or less was considered the threshold for statistical significance.

## Results

### Demography

A total of 1700 physical questionnaires were administered. All valid responses received were 1767, comprising of 321 online and 1446 paper-based responses. Response rate for paper-based sample was 85.06%. Of the 1767 responses obtained, female participants were in the minority as indicated by 42.2% of the sample. Majority (54.9%) of the study participants were between the ages of 18 and 30 years, whilst those above 60 years represented the least proportion of the sample. Also, those with first degree or higher national diploma (HND) represented the most populous proportion of the study participants surveyed, further details on socio-demographic characteristics of the sample are presented in Table 1 below.

**Table 1:** Socio-Demographic Characteristics.

**Variables**	**Frequency (%)**
**Gender**	
Male	1022 (57.8)
Female	745 (42.2)
**Age**	
18 – 30	967 (54.9)
31 – 40	428 (24.2)
41 – 50	239 (13.5)
51 – 60	73 (4.1)
Above 60	57 (3.2)
**Education**	
Primary	45 (2.5)
Secondary	227 (12.9)
National diploma/NCE	260 (14.7)
First degree/HND	950 (53.8)
Postgraduate	284 (16.1)
**Occupation**	
Unemployed	235 (13.3)
Self-employed	386 (21.8)
Private	594 (33.6)
Government sector	454 (25.7)
Retired	44 (2.5)
Others	54 (3.1)

## Factors associated with COVID-19 vaccine hesitancy

**Fig. 1:**
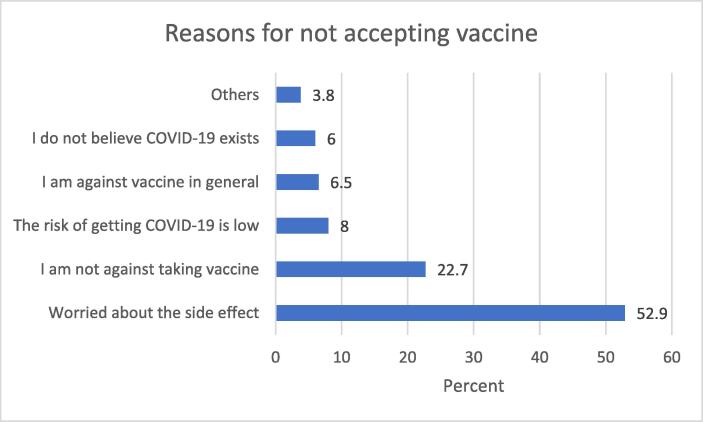
Acceptance of COVID-19 Vaccine.

Identifying factors associated with non-acceptance of COVID-19 vaccine can help government and policymakers develop appropriate strategies to help address COVID-19 vaccine hesitancy. From Fig. 1 below, just under a quarter of the sample (22.7%) agreed that they had no reason not to take the vaccine.

Findings indicated in the figure above revealed that more than half of the study participants reported concerns about side effects, as a factor that may prevent their engagement with the vaccine.

## Vaccine safety and risk concerns

**Fig. 2:**
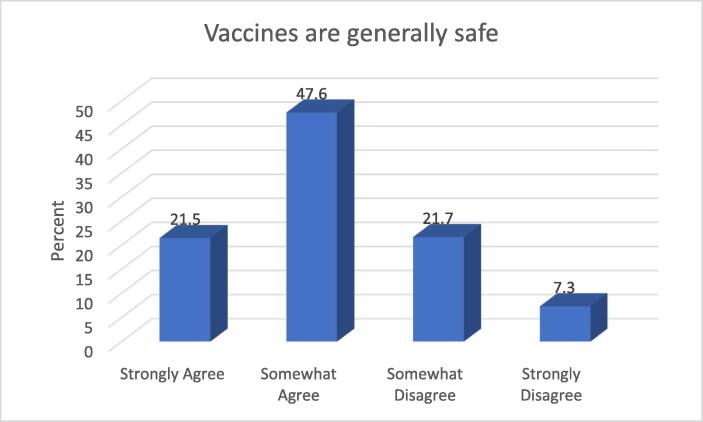
Safety of Vaccines.

Assuring citizens of the safety of vaccines is critical to achieving public health immunisation goals. Understanding population perceptions of product safety is therefore critical to understanding issues around hesitancy. From Fig. 2 below, slightly above two-thirds of the study participants (69.1%) agreed that vaccines are generally safe.

From the findings in this aspect of the study, it appears that a significant proportion of the participants do not generally trust the safety of vaccines. Individuals expect relevant stakeholders such as the World Health Organisation and various regulatory authorities to ensure that they are not subjected to harm during vaccination.

**Fig. 3:**
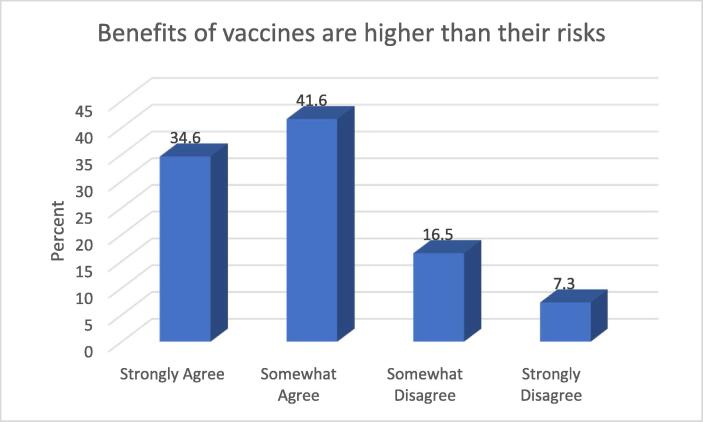
Benefits and Risks of Vaccines.

As with all other pharmaceuticals, vaccines’ approval for use depends on clear evidence that the benefits associated with the interventions outweigh the risks. Since most vaccines typically contain a weakened or killed form of disease-causing organism, it is understandable that concerns may be heightened for this product. Findings in Fig. 3 below shows that about three-quarters (76.2%) of the study participants surveyed indicated that benefits of vaccines are higher than their risk.

The majority of the study sample agreed that vaccines’ benefits outweigh associated risks however, close to a quarter of the population felt otherwise. Understanding vaccines’ mechanism of action with respect to stimulating acquired immunity to infectious disease may aid a better understanding amongst the populace as regards the benefits and risks associated with the product.

## Willingness to pay for COVID-19 vaccination

A total number of 1502, representing 85.1% of the sample indicated that COVID-19 vaccine should be administered at no cost to citizens, whilst 264 participants representing 14.9% indicated that citizens should pay for vaccination. In addition, only 460 study participants representing 26% were willing to pay a fee to be vaccinated against COVID-19.

**Fig. 4:**
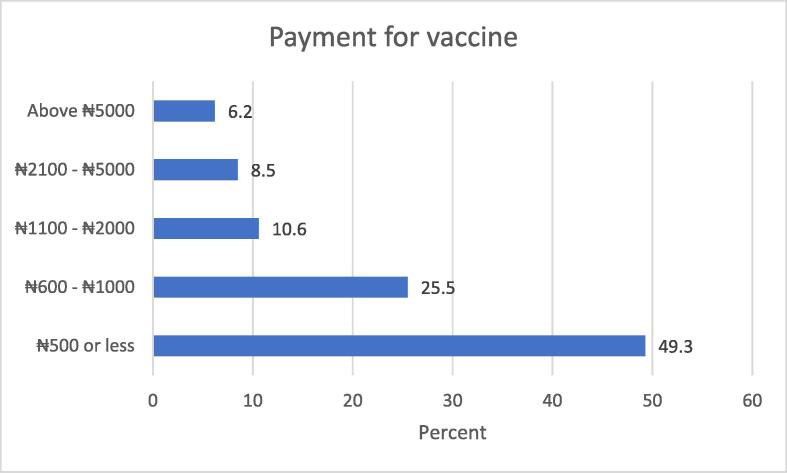
Payment for COVID-19 Vaccination.

Amongst the study participants that indicated interest in paying a fee for COVID-19 vaccination, slightly less than half of them were not willing to pay above five hundred Nigerian naira. Fig. 4 below provides frequency distribution of the maximum cost the participants were willing to pay for vaccination.

This study has provided some evidence of willingness to pay for COVID-19 vaccine, however this disposition was limited by the small proportion that indicated their readiness to pay above five hundred Nigerian naira.

Sequel to the descriptive statistics undertaken, further analyses were undertaken to determine the association between responses of the study participants and their socio-demographic characteristics. Cross tabulation revealed that that 48.0% of the participants that had previously been infected with COVID-19 indicated willingness to pay for vaccination, compared to 25.7% of those that had never been infected. This finding was statistically significant (*p =* 0.012). Other statically significant findings also emerged with respect to respondents’ age, sex and educational background. Older participants were more likely to pay for COVID-19 vaccination, compared to their younger contemporaries (*p* < 0.001); males were more likely to pay for COVID-19 vaccination (*p* = 0.012); and majority of those with only primary education indicated willingness to pay for vaccination (*p* < 0.001). Table 2 below presents details relating demographically differentiated participants’ responses on willingness to pay for COVID-19 vaccination.

**Table 2:** Cross Tabulation of Demography with Willingness to Pay for Vaccination.

**Demography**	**Yes**	**No**	**X^2^**	***p-*value**
**Age**				
18–30	225 (23.2)	743 (76.8)	28.315	< 0.001
31 – 40	119 (27.8)	309 (72.2)		
41–50	58 (24.3)	181 (75.7)		
51 – 60	33 (45.2)	40 (54.8)		
Above 60	25 (43.9)	32 (56.1)		
**Education**				
Primary	23 (51.1)	22 (48.9)	30.067	< 0.001
Secondary school	60 (26.5)	166 (73.5)		
National diploma/NCE	64 (24.6)	196 (75.4)		
First degree/HND	216 (22.7)	734 (77.3)		
Postgraduate	97 (34.2)	187 (65.8)		
**Gender**				
Male	289 (28.3)	733 (71.7)	6.624	0.012
Female	171 (23.0)	573 (77.0)		

## Discussion

Findings from this study indicates that majority of the participants expressed some hesitancy with respect to COVID-19 vaccines, possibly due to perceived side effects. Evidence in the literature suggests that this may be due to the accelerated development of the vaccine [7]. Other contributory factors may include several negative campaigns targeted at discrediting the vaccines and querying its safety [18]. A similar finding was also reported in Israel where majority of participants in that study indicated that they were worried about the side effects of COVID-19 vaccines [19]. This finding was however reported prior to that country’s authorisation and consequent widespread utilisation of the vaccine. The development and commercialization of vaccines usually take more than a decade, especially due to the various activities necessary to ensure short-term and long-term safety and efficacy [20], [21]. However, though the present cohort of COVID-19 vaccines were developed expeditiously, there is little or no evidence that suggests that safety had been sacrificed for speed [22]. Nevertheless, given the accelerated development of these vaccines, concerns expressed in this study are logical and if not properly addressed, could increase hesitancy. A failure to address these concerns could delay or prevent the achievement of herd immunity alongside other possible public health consequences. Although a relatively small proportion of the sample indicated their disbelief in the existence of COVID-19, this finding is still significant, given the ramifications for misinformation together with the consequent risks for public health.

Vaccine hesitancy is not exclusive to the COVID-19 pandemic. In previous pandemics like H1N1 influenza, the acceptance rate associated with vaccines for relevant diseases ranged from 8% to 67% across different countries [23]. Vaccine acceptance has therefore been identified as a complex phenomenon, with contexts varying depending on the time, place and perceived behavioural proclivities of the community under study [24], [25], [26], [27]. In the Chinese setting, demographics and public perception were reported as predictors of vaccine acceptance [28]. Disease specific evidence from Ireland revealed that healthcare workers avoided seasonal influenza vaccination as a result of their misconceptions relating to the efficacy of the vaccine [29]. Further studies in the United States identified effectiveness of vaccine, social influence, and health insurance as key predictors of acceptance for the same vaccine [30]. In the United Arab Emirates, a study that investigated parents’ attitudes towards childhood vaccination reported that only few parents were hesitant towards childhood vaccination [30]. In a similar vein, relevant evidence exists which suggests that people who have had a previous exposure to vaccines were more confident as regards receiving vaccines for another ailment [31], [32]. Providing the population with validated evidence-based scientific information is necessary in order to counter publicly available misinformation efforts currently in circulation.

Regarding general vaccines’ perception, about a third of this study’s participants had concerns about vaccines’ safety, whilst close to a quarter felt that risks associated with vaccines outweighed the benefits. Even though these proportions are in the minority, they are nevertheless worrisome. Historically, there is overwhelming scientific evidence that confirms the general safety and effectiveness of vaccines [33], [34], [35], [36]. Even in specific population groups such as children, adolescents, and adults, the findings from the extant literature support the safety of vaccination as a public health intervention [37], [38]. Although there is evidence that adverse effects may arise from vaccination, they are generally mild [39], especially when viewed in relation to the public health benefits. Given the emergent findings from this study, the need for continuous enlightenment of the public about vaccine safety cannot be overemphasized. Targeted campaigns that highlight simple risk benefit analysis of the interventions can also help improve awareness as well as consequent acceptance.

In this study, majority of the study participants indicated that COVID-19 vaccine should be administered at no cost, and only a quarter of the study participants were willing to pay a fee to be vaccinated against COVID-19. Also, half of the study participants who indicated interest in paying for COVID-19 vaccination were not willing to pay above five hundred Nigerian naira. These findings are contrary to findings from other settings that report a strong willingness to pay for vaccination [28], [40]. Despite the fact that only a quarter of the study respondents indicated willingness to pay for COVID-19 vaccination, a significant percentage of this proportion indicated readiness to pay above the Naira equivalent of $1.90 which is the daily threshold for absolute poverty [41]. Further insights into the correlations between these emergent relationships could help policymakers determine eligibility criteria for immunisation campaigns, particularly for resource scarce settings. The findings of this study however provide some insights for policymakers in a number of critical areas. For instance, government funding remains critical for the immediate stimulation of a robust and comprehensive COVID-19 immunisation campaign. However, if and when out-of-pocket payment is considered, the thresholds identified in this study will aid the articulation of relevant decision making matrices for immunisation selection including possible means testing. Findings from this study also further validate the Medicines’ Security concept which strongly supports local production of pharmaceuticals as a means of ensuring access to high quality and affordable pharmaceutical products [42]. Prioritising the local production of vaccines can lead to an exponential improvement in the access to these critical products, especially with ongoing vaccines nationalism which disenfranchises developing countries such as Nigeria [23].

A strong association was observed between age and willingness to pay for COVID-19 vaccination. Older people were more likely to pay for COVID-19 vaccination. This may be due to existing evidence that associates COVID-19 with more severe morbidity and higher mortality rates in older people [43], [44], [45]. A positive association was also observed between being male and being willing to pay for COVID-19 vaccination. Available evidence suggests higher risks for COVID-19 complications, infectivity, and death among males [46]. This sex-based difference in COVID-19 mortality may have contributed to more males indicating willingness to pay for COVID-19 vaccination. Additionally, the study revealed a statistically significant difference in willingness to pay for the vaccine, based on first-hand experience of the disease. Individuals that had previously been infected with COVID-19 were more likely to pay for vaccination, probably due to their unsavoury experience with the disease. It is therefore logical that this demography would be more willing to expend resources in order to ensure protection from future exposure.

In Nigeria, relevant studies have been undertaken to explore how willing the population are, regarding paying for healthcare interventions, including vaccines [47], [48], [49], [50], [51], [52]. This is however the first study to explore this concept with respect to COVID-19 vaccines. Given the significant health and socioeconomic impact of the pandemic, the emergent findings of this study can provide critical insights for policy and healthcare decision making with regards to COVID-19 vaccination.

## Conclusion

This study identified that concerns relating to safety and side effects constitute factors that could contribute to COVID-19 vaccine hesitancy. To avoid poor implementation outcomes associated with vaccine hesitancy, population concerns about safety should be highlighted and comprehensively addressed during enlightenment campaigns. Also, developing engagement strategies that clearly outline that benefits associated with COVID-19 vaccines outweigh the associated risks, can help better inform the populace and consequently improve their acceptance of the intervention.

Paying a fee for COVID-19 vaccination may reduce uptake of the vaccine as revealed by this study. Since it is critical for government and policymakers to develop contextual strategies aimed at achieving optimal immunisation, this emergent evidence can help improve uptake and reduce hesitancy. The current policy direction suggests government’s responsibility for funding the first phase of the immunisation campaign. If a policy change is being considered, findings relating to willingness to pay from this study can underpin an effective selection framework. Individuals of the male persuasion and being previously infected with COVID-19 were associated with higher willingness to pay for COVID-19 vaccination. Similarly, when compared to their younger contemporaries, older persons were more likely to pay to be vaccinated against COVID-19.

Given the national and global significance of this issue, further studies are strongly recommended in order to enable more robust and comprehensive exploration. Future findings that build on the foundation of this study will be invaluable in developing contextual strategies that address hesitancy and various population concerns, not just for COVID-19, but for other lifesaving vaccines as well.

The limitation of this study has to do with the sampling strategies which may not be a representative of adults in Abuja. However, strengths of the study are the pre-testing of the survey instrument and reasonable sample size used.

## Funding

No grant was received for this research.

## Declaration of Competing Interest

The author declare that there is no known competing financial interests or personal relationships that could have appeared to influence the work reported in this paper.

## References

[1] Di Domenico L., Pullano G., Sabbatini C.E., Boëlle P.Y., Colizza V. (2020). Impact of lockdown on COVID-19 epidemic in Île-de-France and possible exit strategies. BMC Med.

[2] Kumari P., Toshniwal D. (2020 Jun). Impact of lockdown measures during COVID-19 on air quality–A case study of India. Int J Environ Health Res.

[3] Lazarus J.V., Ratzan S.C., Palayew A., Gostin L.O., Larson H.J., Rabin K. (2021). A global survey of potential acceptance of a COVID-19 vaccine. Nat Med.

[4] European Parliament. European Parliament resolution of 19 April 2018 on vaccine hesitancy and drop in vaccination rates in Europe (2017/2951 RSP); 2018. https://www.europarl.europa.eu/doceo/document/TA-8-2018-0188_EN.pdf [accessed 25 January 2020].

[5] Enserink M, Cohen, J. Fact-checking Judy Mikovits, the controversial virologist attacking Anthony Fauci in a viral conspiracy video. Science; 2020. https://www.sciencemag.org/news/2020/05/fact-checking-judy-mikovitscontroversial-virologist-attacking-anthony-fauci-viral [accessed 25 January 2021].

[6] Cornwall W. (2020). Officials gird for a war on vaccine misinformation. Science.

[7] Fadda M., Albanese E., Suggs L.S. (2020). When a COVID-19 vaccine is ready, will we all be ready for it?. Int. J. Public Health.

[8] Fine P., Eames K., Heymann D.L. (2011). “Herd immunity”: A rough guide. Clin Infect Dis.

[9] Laxminarayan R, Jamison DT, Krupnick AJ, Norheim OF. Valuing vaccines using value of statistical life measures. Vaccine. 2014;32(39):5065–5070. https://doi.org/doi:10.1016/j.vaccine.2014.07.003. 10.1016/j.vaccine.2014.07.003 25045822

[10] Harapan H., Fajar J.K., Sasmono R.T., Kuch U. (2017). Dengue vaccine acceptance and willingness to pay. Hum Vaccines Immunother.

[11] Kim S.Y., Sagiraju H., Russell L.B., Sinha A. (2014). Willingness-to-pay for vaccines in low-and middle-income countries: a systematic review. Ann Vaccines Immunization.

[12] Seib K., Pollard A.J., de Wals P., Andrews R.M., Zhou F., Hatchett R.J. (2017). Policy making for vaccine use as a driver of vaccine innovation and development in the developed world. Vaccine..

[13] Wouters O.J., Shadlen K.C., Salcher-Konrad M., Pollard A.J., Larson H.J., Teerawattananon Y. (2021). Challenges in ensuring global access to COVID-19 vaccines: production, affordability, allocation, and deployment. The Lancet..

[14] Ogundele O.A., Ogundele T., Beloved O. (2020). Vaccine hesitancy in Nigeria: Contributing factors–way forward. Nigerian J General Pract.

[15] Goodman, L.A. (1961). Snowball sampling. Annals of Mathematical Statistics. 32 (1): 148–170. https://doi.org/0.1214/aoms/1177705148.

[16] Baltar F., Brunet I. (2012). Social research 2.0: virtual snowball sampling method using Facebook“. Internet Res..

[17] Andersson A. (2011). A systematic examination of a random sampling strategy for source apportionment calculations. Sci Total Environ.

[18] Biasio L.R., Bonaccorsi G., Lorini C., Pecorelli S. (2020). Assessing COVID-19 vaccine literacy: a preliminary online survey. Hum Vaccines Immunother.

[19] Dror A.A., Eisenbach N., Taiber S., Morozov N.G., Mizrachi M., Zigron A. (2020). Vaccine hesitancy: the next challenge in the fight against COVID-19. Eur J Epidemiol.

[20] Calina D., Docea A.O., Petrakis D., Egorov A.M., Ishmukhametov A.A., Gabibov A.G. (2020 Jul 1). Towards effective COVID-19 vaccines: Updates, perspectives and challenges. Int J Mol Med.

[21] Kostoff R.N., Briggs M.B., Porter A.L., Spandidos D.A., Tsatsakis A. (2020). [Comment] COVID-19 vaccine safety. Int J Mol Med.

[22] Polack F.P., Thomas S.J., Kitchin N., Absalon J., Gurtman A., Lockhart S. (2020). Safety and efficacy of the BNT162b2 mRNA Covid-19 vaccine. N Engl J Med.

[23] Larson H.J., Clarke R.M., Jarrett C., Eckersberger E., Levine Z., Schulz W.S. (2018). Measuring trust in vaccination: A systematic review. Hum Vaccines Immunother.

[24] Wilson K., Nguyen H.H., Brehaut H. (2011). Acceptance of a pandemic influenza vaccine: a systematic review of surveys of the general public. Infect Drug Resist.

[25] Larson H.J., Jarrett C., Eckersberger E., Smith D.M.D., Paterson P. (2014). Understanding vaccine hesitancy around vaccines and vaccination from a global perspective: a systematic review of published literature, 2007–2012. Vaccine.

[26] Habersaat K.B., Jackson C. (2020). Understanding vaccine acceptance and demand – and ways to increase them. Bundesgesundheitsblatt Gesundheitsforschung Gesundheitsschutz..

[27] Xiao X., Wong R.M. (2020). Vaccine hesitancy and perceived behavioral control: a meta-analysis. Vaccine..

[28] Chan E.-Y.-Y., Cheng C.-K.-Y., Tam G.-C.-H., Huang Z., Lee P.Y. (2015). Willingness of future A/H7N9 influenza vaccine uptake: a cross-sectional study of Hong Kong community. Vaccine..

[29] Halpin C., Reid B. (2019). Attitudes and beliefs of healthcare workers about influenza vaccination. Nursing Older People..

[30] Alsuwaidi A.R., Elbarazi I., Al-Hamad S., Aldhaheri R., Sheek-Hussein M., Narchi H. (2020). Vaccine hesitancy and its determinants among Arab parents: a cross-sectional survey in the United Arab Emirates. Hum. Vaccines Immunother..

[31] Setbon M., Raude J. (2010). Factors in vaccination intention against the pandemic influenza A/H1N1. Eur J Public Health..

[32] Gidengil C.A., Parker A.M., Zikmund-Fisher B.J. (2012). Trends in risk perceptions and vaccination intentions: a longitudinal study of the first year of the H1N1 pandemic. Am J Public Health..

[33] Orenstein W.A., Bernier R.H., Dondero T.J., Hinman A.R., Marks J.S., Bart K.J. (1985). Field evaluation of vaccine efficacy. Bull World Health Organ.

[34] Ellenberg S.S., Chen R.T. (1997). The complicated task of monitoring vaccine safety. Public Health Rep.

[35] Dean N.E., Gsell P.S., Brookmeyer R., De Gruttola V., Donnelly C.A., Halloran M.E. (2019). Design of vaccine efficacy trials during public health emergencies. Sci Transl Med.

[36] Corey B.L., Mascola J.R., Fauci A.S., Collins F.S. (2020). A strategic approach to COVID-19 vaccine R&D. Science.

[37] Maglione M.A., Das L., Raaen L., Smith A., Chari R., Newberry S. (2014). Safety of vaccines used for routine immunization of U.S. children: a systematic review. Pediatrics.

[38] Dudley MZ, Halsey N A, Omer SB, Orenstein WA, O'Leary ST, Limaye RJ, Salmon DA (2020). “The state of vaccine safety science: systematic reviews of the evidence”. The Lancet Infectious Diseases. 2020;20(5): e80–e89. https://doi.org/10.1016/s1473-3099(20)30130-4. 10.1016/S1473-3099(20)30130-4 32278359

[39] Centre for Disease Control and Prevention. Vaccines & Immunizations. 2020. https://www.cdc.gov/vaccines/vac-gen/side-effects.htm (accessed 19 February 2021).

[40] Harapan H., Wagner A.L., Yufika A., Winardi W., Anwar S., Gan A.K. (2020). Willingness-to-pay for a COVID-19 vaccine and its associated determinants in Indonesia. Hum Vaccin Immunother.

[41] World Bank. Poverty and Equity Data. 2020. https://povertydata.worldbank.org/poverty/country/NGA (accessed 26 February 2021).

[42] Adigwe O.P. (2020). Stakeholders’ Perspective of Role of Policy and Legislation in Achieving Medicines’ Security. Int J World Policy and Dev Stud.

[43] Wu Z., McGoogan J.M. (2020). Characteristics of and important lessons from the coronavirus disease 2019 (COVID-19) outbreak in China: summary of a report of 72 314 cases from the Chinese Center for Disease Control and Prevention. JAMA.

[44] Zhou F., Yu T., Du R., Fan G., Liu Y., Liu Z. (2020). Clinical course and risk factors for mortality of adult inpatients with COVID-19 in Wuhan, China: a retrospective cohort study. Lancet.

[45] Shahid Z., Kalayanamitra R., McClafferty B., Kepko D., Ramgobin D., Patel R. (2020). COVID-19 and older adults: what we know. J Am Geriatr Soc.

[46] Galbadage T., Peterson B.M., Awada J., Buck A., Ramirez D., Wilson J. (2020). Systematic review and meta-analysis of sex-specific COVID-19 clinical outcomes. Front Med.

[47] Uzochukwu B.S.C., Onwujekwe O.E. (2003). Altruistic willingness to pay for family planning services: a study in Southeast Nigeria. Nigerian J Community Med Primary Health Care..

[48] Onwujekwe O., Shu E., Chima R., Onyido A., Okonkwo P. (2000). Willingness to pay for the retreatment mosquito nets with insecticide in four communities of south-eastern Nigeria. Trop Med Int Health.

[49] Uzochukwu B.S., Onwujekwe O.E., Uguru N.P., Ughasoro M.D., Ezeoke O.P. (2010 Dec). Willingness to pay for rapid diagnostic tests for the diagnosis and treatment of malaria in southeast Nigeria: ex post and ex ante. Int J Equity Health..

[50] Udezi W.A., Usifoh C.O., Ihimekpen (2010). Willingness to pay for three hypothetical malaria vaccines in Nigeria. Clin Ther.

[51] Ughasoro M.D., Esangbedo D.O., Tagbo B.N., Mejeha I.C. (2015). Acceptability and Willingness-toPay for a Hypothetical Ebola Virus Vaccine in Nigeria. PLoS Negl Trop Dis.

[52] Umeh I.B., Nduka S.O., Ekwunife O.I. (2016 Dec). Mothers’ willingness to pay for HPV vaccines in Anambra state, Nigeria: a cross sectional contingent valuation study. Cost Effectiveness Resource Allocation..

