# Supplementary material for: COVID-19 vaccine hesitancy and willingness to pay: Emergent factors from a cross-sectional study in Nigeria
Source: Vaccine X. 2021 Sep 3;9:100112. doi: 10.1016/j.jvacx.2021.100112 (PMC8413098; doi:10.1016/j.jvacx.2021.100112)
Supplement: Supplementary data 1 [file mmc1.docx]

**Table 3: Online and Physical Socio-Demographic Characteristics**

| **Variables** | **Online n = 321**  **Frequency (%)** | **Physical n = 1446**  **Frequency (%)** |
| --- | --- | --- |
| Gender |  |  |
| Male | 179 (55.8) | 843 (58.3) |
| Female | 142 (44.2) | 603 (41.7) |
| Age |  |  |
| 18 – 30 | 169 (52.6) | 800 (55.4) |
| 31 – 40 | 81 (25.2) | 347 (24.0) |
| 41 – 50 | 48 (15.0) | 191 (13.2) |
| 51 – 60 | 13 (4.0) | 60 (4.2) |
| Above 60 | 10 (3.1) | 47 (3.3) |
| Education |  |  |
| Primary | 6 (1.9) | 39 (2.7) |
| Secondary | 40 (12.5) | 187 (12.9) |
| National diploma/NCE | 46 (14.3) | 214 (14.8) |
| First degree/HND | 159 (49.5) | 791 (54.7) |
| Postgraduate | 70 (2.8) | 214 (14.8) |
| Occupation |  |  |
| Unemployed | 38 (11.8) | 197 (13.6) |
| Self-employed | 73 (22.7) | 313 (21.6) |
| Private | 105 (32.7) | 489 (33.8) |
| Government sector | 85 (26.5) | 369 (25.5) |
| Retired | 9 (2.8) | 35 (2.4) |
| Others | 11 (3.4) | 43 (2.0) |

**Table 4: Online and Physical Findings on Vaccine Hesitancy and Willingness to Pay for Vaccination**

| **Variables** | **Online n = 321**  **Frequency (%)** | **Physical n = 1446**  **Frequency (%)** |
| --- | --- | --- |
| Reasons for not accepting vaccine |  |  |
| I am worried about the side effect | 173 (53.9) | 760 (52.7) |
| The risk of getting COVID-19 is low | 25 (7.8) | 116 (8.0) |
| I do not believe COVID-19 exist | 24 (7.5) | 82 (5.7) |
| I am against vaccine in general | 22 (6.9) | 93 (6.4) |
| I am not against taking vaccine | 57 (17.8) | 344 (23.9) |
| Others | 20 (6.2) | 47 (3.3) |
| Vaccines are generally safe |  |  |
| Strongly disagree | 41 (12.8) | 122 (8.4) |
| Somewhat disagree | 61 (19.1) | 321 (22.2) |
| Somewhat agree | 154 (48.1) | 685 (47.4) |
| Strongly agree | 64 (20.0) | 316 (21.9) |
| Benefits of vaccines are higher than their risks |  |  |
| Strongly disagree | 28 (8.8) | 100 (6.9) |
| Somewhat disagree | 52 (16.3) | 240 (16.9) |
| Somewhat agree | 134 (41.9 | 600 (41.5) |
| Strongly agree | 106 (33.1) | 505 (34.9) |
| Are you of the opinion that COVID-19 vaccine should be administered at no cost to citizens |  |  |
| Yes | 261 (81.3) | 1241 (85.9) |
| No | 60 (18.7) | 204 (14.1) |
| Would you be willing to pay a fee to be vaccinated |  |  |
| Yes | 84 (26.2) | 376 (26.0) |
| No | 237 (73.8) | 1067 (73.9) |
| What is the maximum cost you will be willing to pay | n =126 | n = 357 |
| ₦500 or less | 67 (53.2) | 171 (47.9) |
| ₦600 - ₦1000 | 29 (23.0) | 94 (26.3) |
| ₦1100 - ₦2000 | 9 (7.1) | 42 (2.9) |
| ₦2100 - ₦5000 | 13 (10.3) | 28 (1.9) |
| Above ₦5000 | 8 (6.3) | 22 (1.5) |
